# Experimental Investigations of the Dental Filling Materials: Establishing Elastic Moduli and Poisson’s Ratios

**DOI:** 10.3390/ma16093456

**Published:** 2023-04-28

**Authors:** Dániel Tamás Száva, Ioan Száva, Sorin Vlase, Andrea Száva

**Affiliations:** 1 Faculty of Dental Medicine, George Emil Palade University of Medicine, Pharmacy, Science and Technology of Targu Mures, 540142 Targu Mures, Romania; 2 Department of Mechanical Engineering, Transilvania University of Brasov, 500036 Brasov, Romania; 3 Romanian Academy of Technical Sciences, 030167 Bucharest, Romania

**Keywords:** dental filling material, DFM, tooth, Poisson’s ratio, Young’s modulus, shearography

## Abstract

The mechanical properties of the dental filling material (DFMs) strongly influence the lifetime and durability of the tooth reparation performed. Among the most significant mechanical characteristics, one has to mention the Poisson’s ratio and the elastic modulus (Young’s modulus). They, during the cyclic mastication load, can prevent or aid in the prevention of secondary dental decays by provoking micro-cracks, the de-bonding of the filling material from the natural dental tissue, as well as fatigue at the level of their interface. The authors performed a scoping analysis of the nowadays-involved experimental methods, together with a critical review, putting in evidence of their advantages and limits. Based on the developments, they propose a new approach in this sense by involving the electronic speckle pattern interferometry (ESPI)/shearography high-accuracy optical method. They illustrate the advantages of this method in establishment of the elastic modulus, but they also propose a high-accuracy methodology in the estimation of Poisson’s ratio. Based on the briefly-illustrated experimental results, one can conclude that ESPI/shearography can become a very useful tool for research, even though it is not a common (nowadays widely applied) method, such as three-point bending or strain gauge methods.

## 1. Introduction

Dental filling materials (DFMs), without reference to their type or to their application manner, are always anisotropic ones. Consequently, their mechanical properties and the corresponding mechanical characteristics depend strongly on the investigation’s direction.

During and after the polymerization of the DFM, due to the produced shrinkage, the inserted amount of DFM produces residual stresses and strains on the interface of the natural dental tissue and this bulk DFM. These local stresses and strains during mastication lead to micro-cracks on these interfaces, which produce the so-called secondary dental decays. Consequently, it is strongly recommended as much as possible to achieve similar mechanical properties for the involved DFM and the natural dental tissue; in this sense, the elasticity moduli as well as the Poisson’s ratio play the most significant role. One other influencing factor is the lateral swelling of the implemented DFM during the mastication, which consists of cyclically applied loads and consequently the DFM’s fatigue, mainly at the level of its interface with the natural dental tissue.

One other issue involves the relatively small dimensions of the inserted amount of the *DFM*.

The above-mentioned facts will determine the behaviors of the suitable experimental investigation methods together with the involved experimental investigation specialist.

Nowadays, the most employable DFMs are the composite type, which, after their insertion into the dental cavity, are subjected to polymerization. The literature considers two distinct phases of the polymerization, i.e., pre-gel and post-gel phases, respectively. In the former, when the inserted DFM is transformed from a viscous liquid into a gel, shrinkage and therefore stress occur as a result. In the latter, when this gel is transformed into a solid material, both shrinkage and stress are produced.

In addition, one other issue, not analyzed in the present survey, consists of interoperability between the natural dental tissue and the implemented (inserted) amount of DFM.

Taking into consideration only the former item (issue), i.e., the experimental establishment of the mechanical characteristics of the DFMs, in the next chapter, the authors offer a critical analysis of the possibly employable experimental methods of the solid body. They present both the advantages and the limits of the nowadays-applied experimental methods; generally speaking, these experimental methods have to be suitable for anisotropic materials as well as for small size specimens.

The authors focus their survey mainly on those experimental methods that can offer the best accuracy in determining E Young’s moduli and ν Poisson’s ratios for a given DFM, utilized/applied for a given patient, but also those not commonly applied by researchers, i.e., mainly non-contact and full-field monitoring methods. From these experimental methods, the authors selected those that fulfill the three major requirements for DFMs, i.e., to be non-contact, full-field methods, as well as to allow for small area monitoring. In this sense, from their point of view, the ESPI/shearography seems to be the best, followed by VIC/DIC and afterwards the holographic interferometry. In Section 2, the readers can obtain other useful details about the analyzed methods.

In this survey, we briefly analyzed several investigation methods, destined for other mechanical characteristics; however, the main objective/goal remains the above-mentioned two parameters, i.e., the Young’s modulus and the Poisson’s ratio. The authors realize that there are several special investigation methods, involved in the DFMs’ evaluation, also very useful, but situated outside of the proposed topic. In addition, how these two parameters (*E* and ν) play a significant role in obtaining a durable and adequate teeth filling is mentioned by most of the cited authors.

## 2. Materials and Methods

From the very large range of literature concerning the experimental methods, the authors selected only those studies which fulfil the above-mentioned requirements (to be non-contact, full field methods as well as suitable for small area monitoring), similar to the analyzed DFMs [1,2,3,4,5,6]. Starting from the experimental methods of the solid body, analyzed as previously mentioned, depending on the dimensions of the investigated area, they can be local or global methods, respectively [7,8]. The former methods, i.e., local ones, offer stress- or strain-state information only at a certain point and along certain directions [1,2,4,8]; the latter are suitable monitoring a large amount of the tested body’s surface, and usually form the visible zone for the observer [3,4,5,6,7,8,9,10,11,12,13,14,15,16,17].

The other classification takes into consideration that even the investigation method either will or will not influence the monitored phenomena when one uses direct-contact or non-contact investigation methods, respectively. The first used methods employ some sensors, fixed on the tested body, and then sensors can take over an amount of the applied load and consequently the stress-strain state of the analyzed body will suffer change [1,2,4,5,6,7,14,15,16,17]. The current methods, being non-contact ones, will not influence in any manner the subsequent phenomena; these are more credible (realistic) [14,15,16,17].

A third classification takes into consideration the offered information, i.e., stresses, deformations, or the corresponding strains, respectively. The former methods are less employable and are relatively difficult, having a lower accuracy too [1,3,4,5,6,7,8,13]. The latter methods are widely employable, with certain accuracy [1,2,3,4,5,6,7,8,9,10,11,12,14,15,16,17].

The last classification involves surface-monitoring as well as volume-monitoring methods. The former category offers only information on the stress or strain state of the monitored surface [4,5,6,8,9,10,11,12,13,14,15,16,17]; the latter provides information on the body’s volume [4,7].

Based on these classifications, for the DFMs, mainly the non-contact and surface-monitoring optical methods with adequate accuracy will be of interest. Nevertheless, taking into consideration the actual developments of the applied investigation methods in DFMs’ mechanical properties evaluation, in the following sections, we analyze the applied methods by different authors.

### 2.1. Electrical Strain Gauge Method (ESGM)

**Figure 1:**
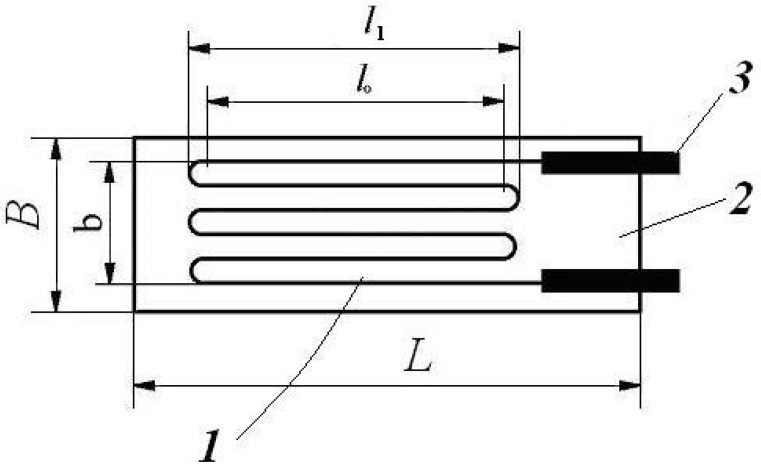
A bonded-wire strain gauge: L—the total length of the gauge; B—the total width of the gauge; $l_{0}$—the wire serpentine’s active length, without the loops length; $l_{1}$—the wire serpentine’s length; b—the wire serpentine’s width; 1—the strain sensing element (here, a metal wire); 2—carrier element; 3—terminals for lead wire connections.

It is a local and direct contact experimental method, offering the local strain state, based on a bonded-wire sensor’s (or bonded-metal-foil sensor, obtained by a photo etching procedure) electrical information changing due to its mechanical loading [1,2,3,4,5,6] (Figure 1).

Based on Lord Kelvin’s observations in 1856, the measurement consists of monitoring the changes in the electrical resistance and the ρ specific electrical resistance of a conductor (metal wire) when its strain state is changed. This means that when the conductor is subjected to tension or compression, as result, its initial $l_{0}$ length is modified and consequently its strain state is too. Together with this strain state variation, its ρ specific electrical resistance also suffers changes, which will be monitored; the obtained electric signal corresponds to the produced strain along the wire longitudinal axis.

The strain

(1) $$\varepsilon \;\left[-\right]=\frac{\Delta l}{l_{0}}\;\left[\frac{m}{m}\right]$$

represents in fact the ratio between the $\Delta l\;\left[m\right]$ elongation (or shortening/contraction) of the initial monitored $l_{0}\;\left[m\right]$ length of the tested object and this initial $l_{0}\;\left[m\right]$ length.

Consequently, a small so-called “strain gauge”, fixed by gluing on the surface of the tested object, monitors during the object’s mechanical or thermal loading the produced strain by means of the ρ specific electrical resistance changing of the strain gauge wire (its grid).

**Figure 2:**
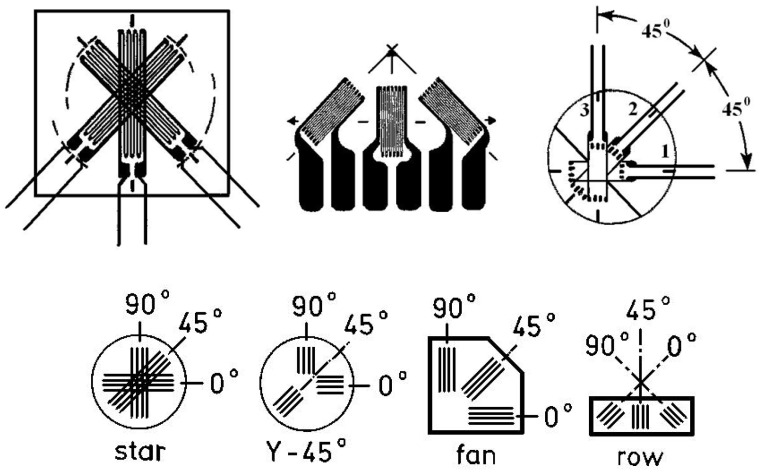
Different technical solutions for the right-angled rosettes [2,3,7,8].

In the case of the anisotropic materials, one has to apply a so-called “rosette”, constituted from three superposed identical grids [2,3,7,8] (wires) (Figure 2).

One has to underline that the stacked arrangement of the rosette’s grids allows for space saving, but, at higher excitation voltage, the heat dissipation can become a serious problem for these strain gauges [2,8]. The accuracy of the method is increasing together with the diminishing of the strain gauge dimensions, more exactly with its $l_{0}$ (the wire serpentine’s active length); it offers locally (in the middle point of the rosette) very precise information on the real strain state of the tested object.

Between its shortcomings, one has to mention the following:

- The rosette type, as well as its material strongly depend on the tested object’s material, i.e., the rosette, as well as its gluing material, are selected in dependence of the tested object’s material;
- This method offers only local information, which is not enough for the anisotropic materials because this kind of material requires whole-surface information instead of local;
- The applied methodology is relatively laborious, requiring highly-qualified people, as well as high-accuracy technology;
- In the authors’ opinion, it does not represent an adequate method for these kinds of tests.

### 2.2. Thin Photo-Elastic Layer’s Technique or Photo-Stress Method (PSM)

The classical photo-elasticity method uses models, instead of the real piece, having the same dimensions, or that are magnified at a desired λ geometric scale. Considering the stresses $\sigma_{p}$ and $\sigma_{m}$, for the prototype and for the photo-elastic model, respectively, produced due to corresponding $F_{p}$ and $F_{m}$ concentrated loads, one has the following relationship to calculate [7]:

(2) $$\sigma_{p}=\lambda^{2}\cdot \frac{F_{p}}{F_{m}}\cdot \sigma_{m}$$

One can underline the useful fact that the obtained stress state does not depend on the $E_{p}/E_{m}$ Young’s moduli (elastic moduli) ratio.

These models, manufactured from some particular, so-called photo-elastic materials, have the special accidental birefringence property. PSM represents one of those particular experimental methods, which offers, directly, the stress-field of the tested object [4,5,6,7,8,13,18]. The sensor element is this special kind of material having an accidental birefringence property. This property means that when the model, manufactured from this special material, is mechanically loaded, it produces the decomposition of the incident light (can resolve it) into two mutually perpendicular component waves, which present a delay in their oscillations (they will present different velocities). The used light source is a special one, i.e., a monochromatic, polarized light, which presents a regular curve path (line, ellipse, or circle) on a normal plane in the light propagation direction, obtained by means of a special optical device, named a polariscope [7,8,13]. Consequently, these light components will emerge from this special material at different times, or there will be a phase difference between them; the obtained light components will present the same directions with the main stresses $\left(\sigma_{1},\;\sigma_{2}\right)$ of the emerging point of the manufactured model.

In the case of the DFMs, only its photo-stress version presents interest, when a thin layer of the photo-elastic material is applied on the tested specimen’s surface.

The tested object, covered with a thin layer of photo-elastic material during its loading, will offer two kind of information (or field of fringes): isoclinic fringes and isochromatic ones [3,4,5,6,7,8,13]. The former offers the $\sigma_{1}$ main stress direction in the analyzed point, i.e., the direction of $\sigma_{1}$ will be tangent to the corresponding isoclinic line, which passes over the analyzed point. The latter represents some geometric locus of the constant main stresses differences $\left(\sigma_{1}-\sigma_{2}\right)$ and consequently of the shear stresses $\tau_{\max}=(\sigma_{1}-\sigma_{2})/2$ in the analyzed point of the model.

**Figure 3:**
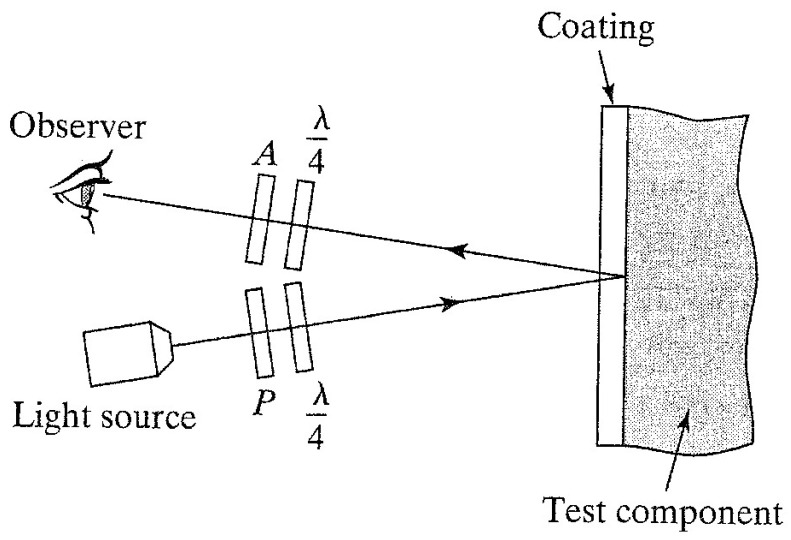
The principle of the reflection polariscope.

In the case of the photo-stress, one uses a reflection polariscope (Figure 3) [5,6,7,18]. The monochromatic light traverses the *P* Polarizer and the λ/4 phase-shifter and arrives through the thin-layer coating to the tested object’s surface. Because the object’s surface is covered with light reflective glue, the light will be reflected and pass through one other λ/4 phase-shifter and the A Analyzer, arriving to the observer, or a CCD camera.

When the λ/4 phase-shifters lock from this optical set, one will obtain the isoclinic fringes, and with the complete optical set: the isochromatic ones.

Based on an accurate calibration, finally one can obtain the corresponding stress field. Unfortunately, the photo-stress method presents several shortcomings such as the following:

- This stress field is in fact a little bit magnified compared to the surface real stress-state ones, due to the fact that the thickness of the layer influences the obtained values by means of the linear σ−ε stress–strain Hooke’s law;
- The method’s accuracy is inversely proportional to the applied layer’s thickness; unfortunately, a greater thickness produces a distortion of the real phenomena by means of a higher stiffness of the subassembly tested piece-thin layer;
- In order to obtain an adequate accuracy, one can produce at least 4–5 fringes, which, for these very small tested specimens, is practically unrealizable;
- The accuracy of the method is much lower than of the forthcoming optical ones, and consequently the authors do not recommend it.

### 2.3. The Moiré-Fringe Method (MFM)

**Figure 4:**
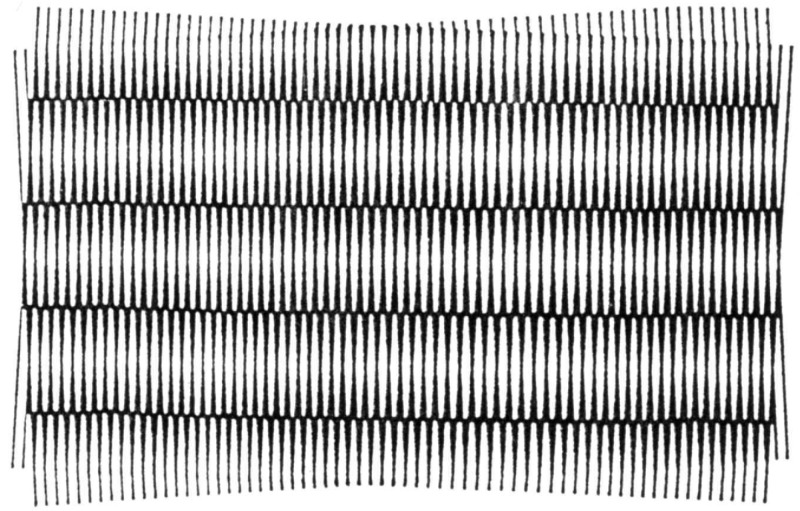
Moiré fringes produced by a small relative rotation of the grids [5].

This method is based on the effect of superposing two similar grates (consisting of parallel, evenly spaced, opaque lines of constant width) that are slightly displaced relative to one another, in that so-called Moiré fringes are produced [7,14,15,16,17] (Figure 4).

There are two main methods [1,4,5,6,7,14,15,17]:

- Geometric Moiré Fringes;
- Shadow Moiré Fringes.

### 2.4. Geometric Moiré Fringes Method

In this case, for the first grid, i.e., reference grating, when kept at a small distance from the object, it will remain un-deformable. The second one, i.e., specimen grating, fixed on the object’s surface will suffer the same deformations as the tested object; consequently, its initial shape and the relative position of the grid’s constitutive lines will be changed. By optical superposition of these relatively displaced grids, one will obtain the so-called Moiré fringes, which offer the displacement state as well as the strain state of the object’s monitored surface. Its accuracy depends on the line spacing; it is mainly suitable for the in-plane deformation field evaluation of the object.

### 2.5. Shadow Moiré Fringes Method

**Figure 5:**
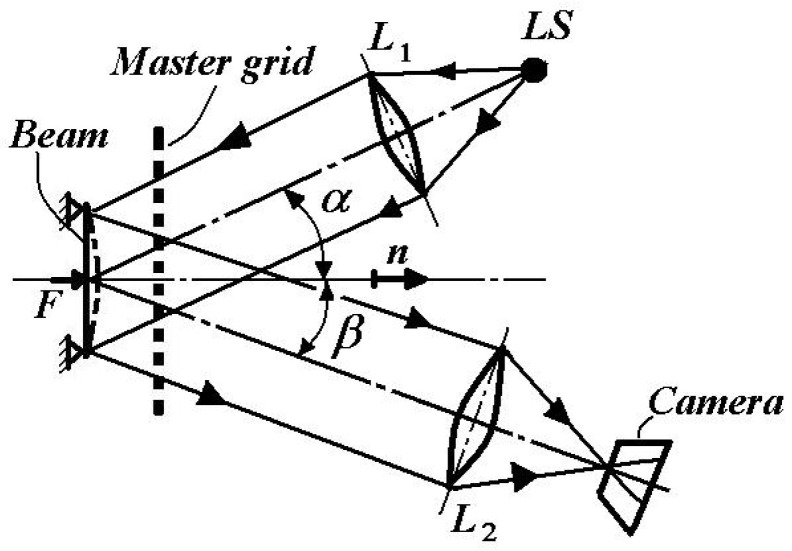
Shadow Moiré optical montage.

The method recommended for the out-of-plane deformations is presented in the optical schema from Figure 5 [1,3,4,5,6,14,15,16,17].

The light, emitted by the LS light-source and collimated by lens *L*_1_, arrives at an angle of incidence *α* to the object (here a beam subjected to bending) through the master grid with pitch p.

The reflected light, at an angle *β* from the tested object, passes through the same master grid and by means of the lens *L*_2_ arrives to the camera. The reflected light beam registers the object’s deformation field by means of the grid’s pitch changing.

The corresponding out-of-plane linear displacement w along the normal direction *n* is [7,19]

(3) $$w=\frac{N\cdot p}{tg\alpha +tg\beta }$$

where *N* is the fringe order; *p* is the pitch of the master grid.

Some advantages of the Moiré methods are the following:

- It is a full-field investigation method, which, generally speaking, is adequate in the composite materials’ investigation when they present either anisotropic or orthotropic behavior;
- Its accuracy, mainly for the relatively large tested bodies, is sufficient;
- It is a universal method using the same equipment for different kinds of materials, and only the involved grids have to be selected adequately;
- This method is relatively cheap and user-friendly.
- The shortcomings of the Moiré methods are the following:
- Its accuracy depends strongly on the pitch *p* of the grid (grids);
- In the case of these small tested specimens, i.e., the DFMs ones, it requires high-density grids, having a very small *p* pitch of the grid (grids);
- It presents lower accuracy than the below-presented holographic interferometry, electronic speckle pattern interferometry/ESPI (or shearography), or video image correlation (VIC) method, respectively;
- The Moiré method, especially for these kinds of tests, will not be very competitive with the above-mentioned optical methods, analyzed below.

### 2.6. The Holographic Interferometry Method (HIM)

At this moment, to the best of the authors’ knowledge, the HIM represents a higher accuracy (12…25 nm) investigation method for general engineering problems [1,3,4,5,6,7,20]. It is a non-contact, full-field investigation method, which is suitable for practically any kind of material (isotropic, orthotropic or anisotropic one), without any special demand on the tested object’s material hardness, starting from hard material (steel, wood, concrete, etc.) up to live tissues too.

**Figure 6:**
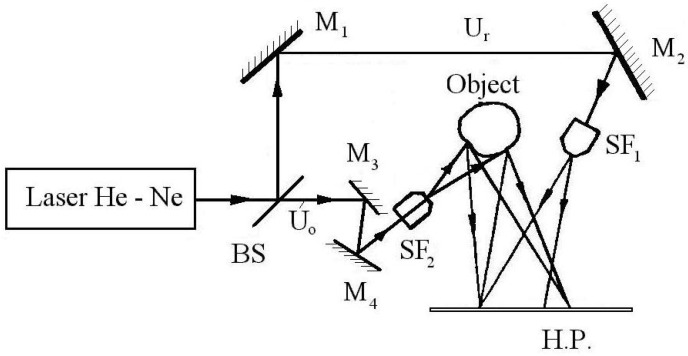
The optical montage of the transmission holograms.

Figure 6 shows the optical principle of the transmission holograms. The monochromatic light beam emitted by a HeNe laser and divided into two parts by means of the beam splitter BS became a reflected part, named reference beam U_r_, and a transmitted part, named object beam U_o_. The reference beam guided by the mirrors M_1_, M_2_ passes through the spatial filter SF_1_ and arrives to the holographic plate HP. The object beam by means of mirrors M_3_, M_4_, and spatial filter SF_2_ arrives to the object. Due to the object’s surface irregularities, which act as secondary mirrors, the beam will be reflected to the holographic plate where it interferes with the reference beam. By their interference, one will obtain a virtual 3D image of the object’s visible surface, named a hologram. If one splits the exposure time into two parts—one for the unloaded state (or preloaded one) of the tested object and one other corresponding to the loaded state—by normal photo-processing, this will result in this type of virtual 3D image, i.e., hologram, where one can observe several interference fringes, which represent the geometric locus of the equally-displaced points of the object’s surface. The use of a relatively complex mathematical evaluation [20] finally results in the displacement field, as well as the corresponding strain field of the surface.

It can be mentioned that the analyzed body has to present diffusely scattering surfaces. This is necessary to obtain for each point in order for the significant property to be able to work as a small secondary light source and to consequently reflect the incident light.

Even if HIM has several advantages, there are some significant shortcomings, namely:

- The environment, where the optical montage is located, requires a very rigorous vibration insulation;
- In order to obtain the requested displacement field, as well as the corresponding strain field, the fringe evaluation is a relatively difficult and time-consuming process;
- The involved persons have to be highly qualified, and the optical system as well as the consumable are relatively expensive.

Due to this, the HIM is recommended to be applied mainly for basic research, which in the case of the DFMs is not entirely truthful. In this sense, based on the authors’ long-term experience, they do not recommend it for DFMs testing.

### 2.7. The Brittle Coating Method (BCM)

This experimental method offers the direction of the $\sigma_{1}$ main stress along the surface of the tested object [1,4,6,7]. In this sense, the tested object’s surface will be covered with a special thin (only several microns) lacquer coat, which for a nominal $\sigma_{0}$ tensile stress will be cracked.

**Figure 7:**
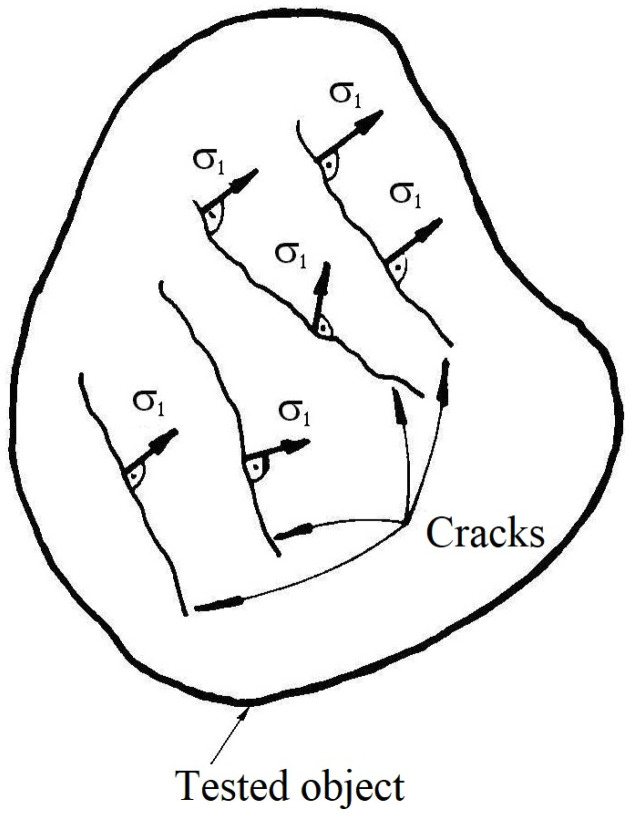
The principle of the Brittle Coating Method.

During a systematic loading of the object, one will monitor (e.g., using a high-resolution CCD camera) the evolution in time of the cracks as well as their propagation directions. So, it became possible to evaluate both the stress concentration loci and their magnitudes. In Figure 7, the principle of the method is presented, where one can observe how the $\sigma_{1}$ main stresses are disposed perpendicular to the cracks; of course, the $\sigma_{2}$ will settle down along the cracks’ directions.

Between the main advantages of the method, one can mention the following:

- It is a full-field method;
- It is relatively cheap;
- The involved persons do not need to be highly qualified.
- Concerning the shortcomings of the method, one can mention the following:
- It is a relatively low-accuracy method;
- Usually, it can be involved as a preliminary investigation method for some more accurate ones, such as the Electrical Strain Gauge Method (for the precise orientation of the strain gauges) and Photo-Stress Method (for a precise location of the isoclinic lines), respectively;
- It can be applied mainly for objects having relatively large tested surfaces, otherwise the produced cracks become superposed;
- It is not recommended for small objects such as the DFMs specimens.

### 2.8. The Video Image Correlation Method (VICM)

The *VICM* represents a very powerful and accurate full field, optical, non-contact investigation method in surface displacement as well as its strain field evaluation [21,22]. Depending on the manufacturing company in the literature, one can find also the digital image correlation—DIC term.

**Figure 8:**
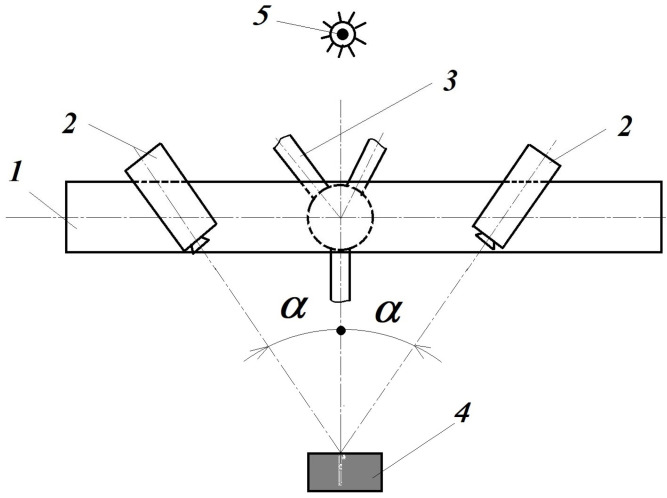
The VIC-3D block diagram: 1—rigid beam (holder); 2—CCD camera; 3—stable trivet; 4—tested object [11,12]; 5—lighting source.

**Figure 9:**
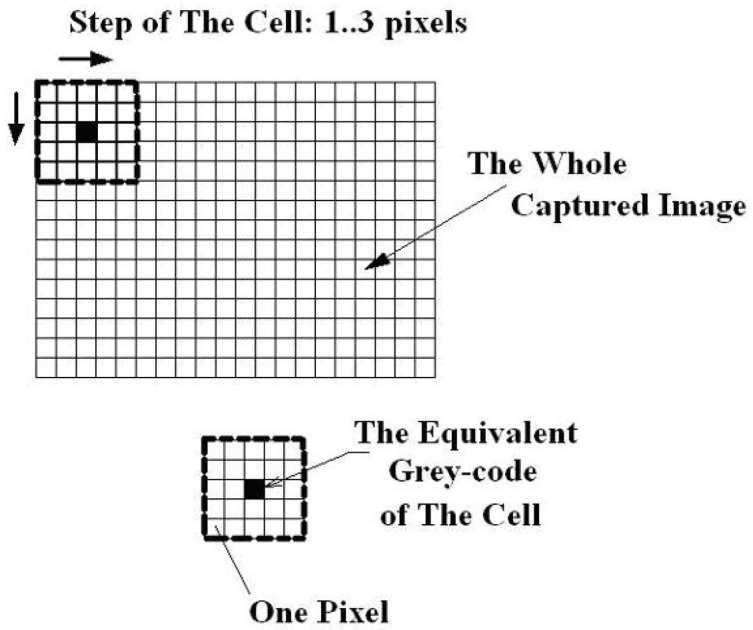
The block schema of the measuring principle [5].

In the block diagram of its optical setup (Figure 8), the two CCD cameras are disposed symmetrically, focused on the surface of the 4 tested object. This optical montage of the two CCD cameras is fixed on the 1 rigid beam, located on the 3 stable trivet. The 5 lighting source is a common white light one, located in the opposite site of the tested object. Its accuracy in displacements for common CCD cameras is lower than 30 nm (nanometers) for a tested surface up to 0.25 m^2^. In advance, the tested object’s surface is sprayed with a water-soluble paint in order to obtain a non-uniform dotted surface; the sizes of dots (speckles) depend on the surface sizes. In this way, one can assure different grey intensities of each pixel from the analyzed surface on the captured images by the two CCD cameras. With a special target, rotated in horizontal and vertical planes on the expectant object’s surface, the sets of these CCD cameras are calibrated. During the measurements, the first set of captured images contains the un-loaded (or pre-loaded) state of the object; after that, the image pairs correspond to its progressively loaded state. The measurement, i.e., the evaluation of the captured image-pairs, consists of a preliminary analysis of them in the following manner [5] (Figure 9).

Each captured image by these CCD cameras contains m⋅n pixels. The first step consists of selecting the area of interest from the captured image, as well as of the so-called subset (primarily cell, or cell) and its scavenging step in the horizontal and vertical directions along the image in order to run through the whole image. In the example from Figure 9, the subset has 5⋅5=25 pixels sizes, and the scavenging step magnitude: 1…3 pixels, respectively.

A final calibration step consists of the identification of a single preselected point from the left and right captured images, and after that the software will perform the predicted analysis.

These calculi of analysis consist of the evaluation of the median pixels’ displacement vectors (more exactly, their x, y, z projections) of the corresponding strains.

Between the main advantages of the *VICM* one can mention the following:

- Because it is a non-contact method, do not influence the analyzed phenomena;
- The same equipment can be used without reference to the material;
- The *VICM* allows for testing both in laboratories and working conditions with the same accuracy.

Concerning the proposed DFMs testing, one of the main shortcomings of the *VICM* consists of fact that it is necessary to use some supplementary optical components, which allow for the monitoring of a small area of interest (only 2–3 cm^2^).

### 2.9. The Electronic Speckle Pattern Interferometry Method (ESPIM)

The last analyzed investigation method is the electronic speckle pattern interferometry (ESPI) or shearography [4,5,7,9,10]. This method, even accepting its little disadvantage of a lower accuracy (“only” 30…40 nm, instead of the 12…25 nm from HIM), substitutes the HIM, due to its ability to capture images also in working environmental conditions.

**Figure 10:**
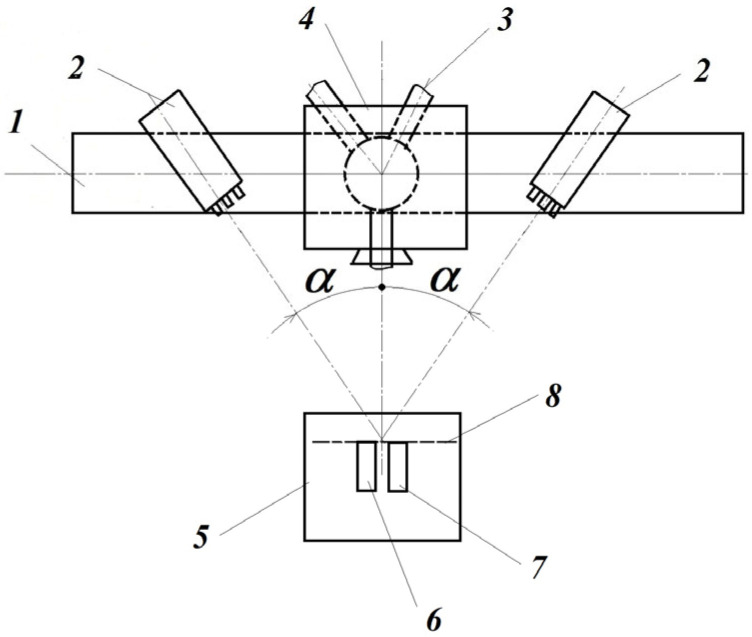
The block schema of the measuring principle: 1—rigid beam; 2—laser diodes; 3—stable trivet; 4—Michelson interferomer; 5—rigid table; 6—tested object; 7—reference boy; 8—plane.

The block schema of the ESPI system, presented in Figure 10, offers a few images on its working principle.

The 3 stable trivet has the 1 rigid beam mounted on it, and these are disposed symmetrically. The sets of the 2 laser diodes, as well as the 4 Michelson interferometer are disposed symmetrically too. The laser diodes are focused on the 8 common plane of the 6 tested object’s surface of the 7 reference body’s ones, respectively; they are disposed on the 5 rigid table. The tested body will be loaded, but the reference one remains unloaded during the experiments.

Both the 6 object and the 7 reference body have to present diffusely scattering surfaces. This is necessary to obtain for each point, as this is a significant property to be able to work as a small secondary light source and consequently reflect the incident light.

In this case, the 4 Michelson interferometer contains not only the usual optical interferometer, but also a high-accuracy CCD camera too. When tilting the shear mirror with a small angle, two points from the object’s surface are superposed in a single point on the focal plane, which, in our case, will be in fact the CCD camera’s image plane.

Depending on the magnitude of overlap of the above-mentioned object’s points, one can speak about shearography (for relatively small amounts) or electronic speckle pattern interferometry with the reference plate method (corresponding to relatively large overlapping).

The phase shift mirror, formed by means of a small piezo-electric element, allows us to obtain, during the exposure, three or four different phase-delayed images, i.e., light path changed images, necessary for an adequate analysis of the captured images.

Without a detailed analysis, one can mention that by using multiple intensity measurements with actively altered light path changes for the partial beam, one can determine the relative phase length for the respective pixel (in the image plane of the *CCD* camera) in regards to the interference phenomenon. From this information, finally one can obtain high-accuracy displacement, as well as the strain field of the monitored surface of the object.

In order to perform a measurement for the first time, by means of the shear mirror, one will ensure a predefined super-positioning (overlapping) of the reference body over the tested object. Afterward, one has to perform a registration (a set of three or four images, captured automatically by the system during a single so-called “exposure”) with the initial state (unloaded or preloaded) of the tested object.

The captured sets of images finally offer the desired displacement and strain fields of the tested object due to its loading. The concrete image evaluation consists of four steps, i.e., the relative phase distribution obtaining, filtering, demodulation, and the final evaluation of the displacement (strain) field, respectively.

Between the main advantages of ESPIM one can mention the following:

- It allows for a high-accuracy evaluation of the whole analyzed object’s surface;
- The method allows for high-accuracy measurements not only in the laboratory, but in working conditions too;
- Being an optical, non-contact method, the same optical setup can be applied for different kinds of materials;
- A disadvantage at the first go-off, i.e., allows only relatively small displacements (half of the incident light’s λ=632.8 nm for the red laser diodes), in the case of the DFMs’ testing became in fact a great advantage; here, both the tested specimens’ sizes and the applied loads are small and consequently the displacements will also be very small.

- In the authors opinion, it seems to be the most suitable common testing method.

In Table 1, based on the below-mentioned references, as well as the authors own practice, we offer a brief synthesis of the analyzed investigation methods, together with their advantages and limits.

## 3. Dental Filling Materials Mechanical Characteristics

The authors of the present survey should discuss the results and how they can be interpreted from the perspective of previous studies and the working hypotheses. The findings and their implications should be discussed in the broadest context possible. Future research directions may also be highlighted.

One of the major goals of the dentist consists of a high-accuracy evaluation of the forces and of the corresponding deformations (strains) during mastication. This kind of evaluation refers not only to subassembly tooth-DFM, but also to DFM as an individual body. The strain of the DFMs to the cyclic (repetitive) action of the mastication force decides whether that material is or is not as adequate as DFM.

The authors of [18] analyzed the effects of the applied mastication force on the stress-strain state of the subassembly natural tooth-DFM. Depending on the place, direction, and nature (statically or dynamically loads) of the mastication force, it is in the range of 200…2440 N. This relatively wide range of the real-obtained mastication force was caused by loading directions as well as the load variation in time, i.e., statically or dynamically applied ones. Their effect on local deformation as well as on strain arises together with the diminishing of the area where they are distributed. In addition they can produce tensile, compression, shear, and composed loads. Depending on the investigated zone of the tooth, and the type and form of the decay of the applied load (mastication force, mainly its direction), either of these solicitations can become predominant. It is very important to consider the shape and the size of the contact zone between the natural tooth and DFM, as well as the decay shape too. In this contact zone, the micro-cracks and de-bodings of the DFM from the natural tissue of the tooth are produced. Supplementary to the composite DFMs, the polymerization also produces shrinkage and internal stresses, which will strongly influence the further deterioration of the filled tooth.

The authors used a special type of microDIC system, which allowed for the monitoring of a small area of interest, consisting of the significant parts of the tested molars. The finite element (FE) simulation, fitted with the experimental results, offers, for further investigations, a very stable database.

Additionally, the authors involved also a micro-computer tomography scanning method in order to perform a better validation of the *FE* models.

They put in evidence the major influence of the shrinkage on the internal contraction stresses state, on the interfacial defects (such as separations), as well as on the micro-leakage in the weak-bonded areas.

In the reference [24], the authors performed, for the amalgams used as *DFM* for extracted premolars, an exhaustive comparison of the fracture resistance values of the most applied preparation methods. The prepared specimens were subjected to axial compression and were monitored for the corresponding fracture loads.

The authors of [25] performed an exhaustive analysis on the bite-force (occlusal force) experimental and numerical evaluation during the mastication. In this sense, they analyze not only the mono-axial force evaluation methods, but also the 3D measuring techniques too. One other useful and significant contribution of the authors consists of the analysis of the bite-force numerical modelling.

In [26,27], the authors present a meticulous and very useful review on the resin composites involved in DFMs’ manufacturing describing the actual developments concerning the involved materials, their obtained technologies, the corresponding mechanical, physical, biological, as well as functional properties, in order to serve as a solid database for further DMFs development. They put in evidence the reciprocal influences between these properties, in order to a better understand the real requirements for these new DFMs, as well as to improve their properties. In addition, the Young’s modulus magnitude of the DFMs is significant, as it has to be closer (in their magnitude) to the natural dental materials.

In [28], the authors describe a strain gauge-based method for composite materials’ contraction monitoring during the polymerization. The described approach proved to be a very suitable real-time monitoring method.

The authors of [29], based on the methodology of the contribution [28], performed an exhaustive study on the effect of delayed photo-activation on the mechanical properties of the dual cured resins (with photo processes as well as chemical curing processes), analyzing the Young’s elastic modulus, the Knoop hardness, as well as the post-gel shrinkage of these resins, which were cured in two stages. The finite element modelling, based on the performed exhaustive experiments, offered useful information on the further dental restoration techniques of the authors.

In [30], the authors analyze the effect of the endodontic treatment procedures on canal shape and the mechanical properties of a tooth, and discuss the parameters and the biomechanical principles of root canal-treated teeth, taking into consideration the mechanical properties of the involved DFMs, between others of the composite resins type.

The authors of [31] analyze four hybrid composite DFMs’ biomechanical properties, i.e., Vickers micro-hardness, compressive strength, direct tensile strength, water absorption, as well as water solubility/degradation. In this survey, mainly the compressive and tensile strengths evaluation methods were interesting. One has to mention that the obtained values were in good correlation with the literature reported ones.

In [19], the authors present a new approach (a modified two-steps incremental technique) in order to improve the marginal adaptation of the composite DFMs, and they also developed a suitable mathematical model for this phenomenon. The presented approach can be a very useful complementary part of an adequate mechanical testing procedure.

The authors of [32] started a search study on the resin-type composite materials, taking into consideration the soft drinks’ undesired effect, i.e., their erosion influence on the DFMs. The specimens, immersed in some adequate acidic drinks in advance, were subjected to three-point bending in a common testing machine. The authors monitored the applied load up to failure, obtaining finally the corresponding flexural strength, as well as the elastic modulus. They provided evidence of this undesired influence, which affects, in a different manner, the analyzed DFMs.

In [27], the authors describe the results of their exhaustive experimental investigations concerning the determination of the static and dynamic elastic moduli (based on impulse excitation method) for 34 resin-based DFMs. The obtained values show the great influence of the elastic module’s value on the de-bonding phenomena.

The authors of [33] performed a comparative analysis of different types of DFMs, as well as the filling depth on the stress-strain state of the first and second molars from a real patient by means of finite element modelling.

In [23], the authors described a useful, full-field, non-contact high-accuracy approach to assess the polymerization shrinkage magnitude for four commercial composite DFMs by applying the in-plane DIC/VIC method with a single CCD camera. The authors obtained useful conclusions on the analyzed DFMs’ shrinkage properties.

The authors of [34] analyzed three original (self-conceived) composite DFMs, by comparing their physical, chemical, as well as their mechanical properties with other four commercial composite DFMs, such as the compressive strength, direct tensile strength, water absorption, as well as water solubility/degradation. The obtained values for their original DFMs were very promising and recommendable for load-bearing surfaces from the posterior teeth.

In [35], the authors analyzed the mechanical properties of some common monomers used in restorative dental composites, such as their flexural modulus of elasticity as well as the corresponding strength, obtained by three-points bending in a common testing machine. By a statistical evaluation, they obtained accurate information on the tested materials.

The authors of [36] performed an exhaustive analysis on the polymerization shrinkage effects’ evaluation. In order to measure the polymerization volumetric shrinkage, the authors presented several experimental methods and the corresponding devices such as the mercury dilatometer, the bonded-disc method, electrical strain gauge method, and the laser speckle-correlation ones, respectively, from [37,38].

The authors of [39] performed a scrupulous investigation in order to establish the involved mechanical characteristics of different DFMs, namely the Young’s modulus, the bulk modulus, the shear modulus, as well as the corresponding Poisson’s ratios. These investigations were performed by means of the classical testing methods, i.e., common axial-compression ones, using both the free lateral swelling (for the Young’s modulus) as well as the radial-constricted cases (for the bulk modulus) too. Calculations via the well-known equations of the theory of elasticity provided the remaining two parameters (the shear modulus, as well as the Poisson’s ratio). The obtained values were in very good correlation with the literature reported ones.

In [20] the authors performed, for two different polymerization shrinkage strains, the equivalent Young’s modulus determination by means of the so-called dental restoration ring method. In addition, in order to establish by experiment the linear shrinkage strains, they applied the bonded disk method. The obtained results were in very good agreement with the literature reported ones.

The authors of the reference [40,41] performed accurate experimental investigations on three types of nano-composites, widely used as DFMs, in order to verify the effects of the polymerization conditions on the Young’s modulus’ and the conversion degree’s magnitudes.

In the reference [42], the authors proposed investigating the influence of the storage conditions (wet and dry) as well as the involved testing method on the obtained Young’s moduli and Poisson’s ratios for the three composite DFMs, subjected to common axial compression tests with free lateral swelling. They concluded that the main causes of the obtained differences consisted of the different test methods, sample dimensions, as well as storage conditions and their preservation time.

## 4. Discussion

In the previous chapter, by performing a development analysis, several experimental methods were considered, as well as evaluated.

From the analyzed methods, one can provide evidence of the three-points bending, performed on rectangular cross-sectional specimens, obtaining finally both the elastic modulus and the corresponding fractural/breaking strengths. Between the main shortcomings of this approach, one can mention the following:

- The authors realize that there are several special investigation methods involved in the DFMs’ evaluation, also very useful, but situated outside of the proposed topic.
- In the authors’ opinion, the selected experimental method has to be non-contact-, full-field-, as well as adequate for small-investigated areas, such are the applied teeth-fillers.
- They focus their survey/evaluation mainly on establishing, as accurately as possible, the Young’s modulus and afterwards the Poisson’s ratio for the DFMs in some local laboratories, which are involved in finding the optimal DFMs for a given patient’s case by fruitful co-operation with dentists. These experimental investigations have to be performed mainly for small size specimens. This last demand, i.e., the testing of small size specimens, will ensure tests with closer dimensions to the real dental filling specimens.
- In addition, how these two parameters (the Young’s modulus and Poisson’s ratio) play a significant role in obtaining a durable and adequate teeth filling is mentioned by most of the cited authors.
- One can mention that the ISO 4049—standard’s recommendation is very comfortable for common research laboratories, where one can find, without any difficulty, a tensile-compression testing machine; this standard concerns only polymer-based restorative materials.

Unfortunately, in the authors’ opinion, there are several shortcomings of the ISO standard’s approach, which supposes three-point bending tests, such as the following:

- Its accuracy is lower than the proposed ESPI’s one, where, without any supplementary devices and difficulties, it can obtain a 10…15 nanometer accuracy.
- The obtained values refer to a global approach, i.e., corresponding to the global behavior of the tested specimen, in contrast with the ESPI, as well as DIC/VIC methods, where became possible to systematically analyze (at different levels and points of the tested specimen) the material response to the applied load, and finally to establish a more suitable magnitude both for Young’s modulus and for Poisson’s ratio, which is proposed in Section 5, Conclusions.
- Taking into consideration the well-known fact that the common tensile-compression testing machines had not foreseen, with high-accuracy displacement-monitoring systems, the obtained displacement-force curves, this means that afterwards the strain–stress curves were not very accurate.
- The common displacement transducers, attached to these testing machines, monitor the global displacement, without any particular reference to the tested specimens’ real (local, i.e., point-related) displacement.
- Even though the testing machine is foreseen with an optical displacement transducer (the so-called video-extensometer), its accuracy corresponds only to the 2D VIC/DIC systems, which is much lower than for the common 3D VIC/DIC systems and far lower than the ESPI’s accuracy.
- Consequently, in both above-mentioned cases, the involved testing machine offers lower accuracy displacement information than the mentioned ESPI/shearography system, which represents in the authors’ opinion the best choice.
- In addition, based on the Strength of Materials, one other significant shortcoming of this type of testing (three-point bending) consists of the coexistence of the undesired shear with the desired bending, which can significantly modify the obtained results.
- A better (more accurate) solution can be the four-point bending, where, in the mid-zone of the tested beam (between the two applied forces), one has only pure bending without shear force; unfortunately, in this case, both a longer tested specimen (in order to allowing positioning of the two force-applying pushing elements) and a more accurate monitoring system will be necessary, which allows for the displacement monitoring in a very small area (between these two force-applying pushing elements).
- Additionally, the involved beam-shape specimens, subjected to bending, are far larger in dimension (their length) than the real teeth-filling bulk material; their response to the applied load is a little bit different to that of the mentioned bulk materials.
- In addition, one can mention that at this moment, the VIC/DIC systems, with their 3D Micro-DIC version, e.g., ISI-Sys GmbH Company from Germany, can offer a new high-accuracy approach, having a field of view of 4.2 × 3.5 mm^2^ with 1.75 μm/pixels accuracy [43], which is much better than the common testing machines’ information.

In the case of the described compression tests, one has to underline as a general shortcoming that they offer only global information on the tested cylindrical specimens, which, as was mentioned above, is not the proper (the best) approach in the case of the anisotropic materials.

In order to determine the demanded ν Poisson’s ratio, the involved sensors, which monitored the lateral swelling, offered also only local information, not along the whole length of the tested specimen. These sensors can be even electronic ones, mounted in a given point of the tested specimen, or an optical extensometer (video-extensometers), annexed to the modern tensile-compression testing machine. These optical extensometers unfortunately have only an accuracy comparable with a 2D-VIC system, i.e., much lower than the aforementioned ESPI/shearography system. The same shortcoming appears in the tensile tests too. Consequently, the resulting ν Poisson’s ratio can be a little bit far from the real value.

One other relatively widely used experimental method was the electrical strain gauge one. This method, being a local (point-attached) one, only in the applied point will offer information. In order to apply it efficiently for an anisotropic material, it will be better (recommended) to use, instead of a single-grid strain gauge, one rosette-type one.

The main shortcomings of this method are the following:

- In order to offer strain state along the tested specimen it can be applied as strain gauges-chain [2,4,7,8], where, along a relatively short active length, there are photo-etched 5…10 miniature strain gauges, each of them with their own electrical wiring; each of these miniature sensors will offer, step-by-step, the corresponding strains.
- The above-mentioned miniature sensors are able to offer information with a step about 2…4 mm, which mainly, in the case of an anisotropic material, does not represent the best way/approach;
- Additionally, these miniature sensors have to be at least the two-elements type, i.e., rectangular rosettes, named also X-rosettes;
- It is not highly recommended for the monitoring of a solidification process, such as the polymerization one.

Some experimental investigations were performed by means of the Moiré fringe method, but, as was mentioned in the previous chapter, this is not the most suitable method due to its relatively low accuracy.

The *DIC*/*VIC* method was applied mainly for the shrinkage as well as contraction stress monitoring during the light curing of some composite DFMs. In this sense, there were some special micro*DIC* systems involved, which allowed for small area monitoring. The system is a relatively expensive and special one, which can be involved only in such kinds of experimental investigations, and consequently not for general purpose measurements. It means that only an effective argument can justify DFMs’ testing. One other approach to the same problem was solved by means of a 2D-DIC system, which presents a relatively low accuracy in comparison with the classical 3D-DIC system.

The laser speckle-correlation method was involved in polymerization shrinkage monitoring (both stresses and contractions). The method is an efficient and high-accuracy optical non-contact one. Unfortunately, it was not applied, as far as the authors know, for the classical mechanical characteristics evaluation (determining), such as with tensile or compression tests analysis, respectively, or elastic moduli or Poisson’s ratios.

Based on the above-mentioned arguments as well as on the authors previous experience, they state that in this kind of testing the most suitable approach can be the ESPI/shearography Method, which incorporates practically all of the required (desired) advantages.

In this sense, it ensures for the small area of monitoring a better accuracy, together with the facilitation of common working conditions; the influences of the environmental vibrations, i.e., the rigid body motions, are automatically eliminated from the measurements results.

How the damages such as micro-leakages as well as micro-fractures of the DFMs strongly depend on the mechanical properties, as well as others from their E elastic module (Young’s module) and the corresponding ν Poisson’s ratio has been demonstrated by several international research studies.

Based on the strength of materials, by definition, the ν Poisson’s ratio represents the ratio between the $\varepsilon_{t}$ transversal strain and the $\varepsilon_{l}$ longitudinal one:

(4) $$\nu \;\left[-\right]=-\frac{\varepsilon_{t}}{\varepsilon_{l}}\;$$

The sign (−) refers to the fact that the longitudinal and the transversal strains will be produced (act) in opposite directions (when the $\varepsilon_{l}$ longitudinal strain is positive, then the $\varepsilon_{t}$ transversal will be negative and vice versa).

**Figure 11:**
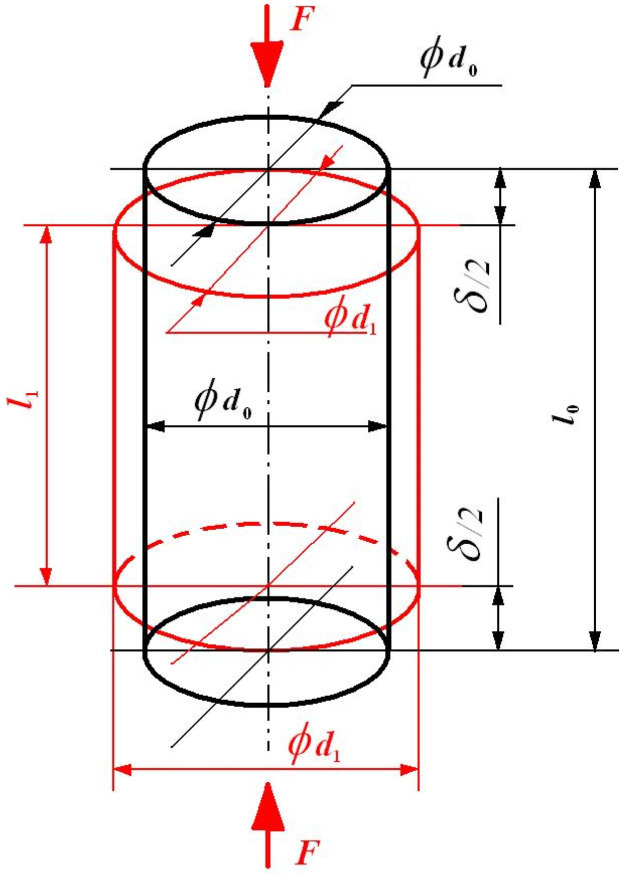
The tested specimen (in black) and its deformed state (in red).

In Figure 11, for the proposed tested specimen, the initial and its final deformed states are provided, which serve in the ν Poisson’s ratio definition.

The initial cylindrical specimen (in black), with $l_{0}\;\left[\mathrm{mm}\right]$ length and $d_{0}\;\left[\mathrm{mm}\right]$ diameter, during its axial compression by force $F\;\left[N\right]$, which in the following will represent in fact the mastication load, suffers not only a $\delta_{l}=\delta =(l_{1}-l_{0})\;\left[\mathrm{mm}\right]$ longitudinal shortening ($\delta_{l}\;\langle \;0$), but also a $\delta_{t}=\delta_{r}=(d_{1}-d_{0})\;\left[\mathrm{mm}\right]$ transversal elongation (swelling), with $\delta_{t}\;\rangle \;0$. 

Finally, one will obtain a shortened cylinder (in red) having $l_{1}\;\left[\mathrm{mm}\right]$ length and $d_{1}\;\left[\mathrm{mm}\right]$ diameter.

The corresponding strains are

- The longitudinal strain
  (5) $$\varepsilon_{l}=\frac{\delta_{l}}{l_{0}}\;\left[-\right]\;,$$

- The transversal strain (in fact the analyzed swelling)
  (6) $$\varepsilon_{t}=\frac{\delta_{t}}{d_{0}}\;\left[-\right]\;.$$

Their ratio, based on Equation (4), offers the wanted (in demand) ν Poisson’s ratio. Meanwhile, due to the applied F load in the tested cylindrical specimen, one produces a normal (axial) stress

(7) $$\sigma =\frac{F}{A}=\frac{F}{\pi \cdot d_{0}^{2}/4}\;\left[\frac{N}{mm^{2}}=\mathrm{MPa}\right]$$

By increasing, step-by-step, the applied F load and monitoring the produced $\delta_{l}$ shortening, one determines with Equation (5) the corresponding $\varepsilon_{l}$ longitudinal strain.

**Figure 12:**
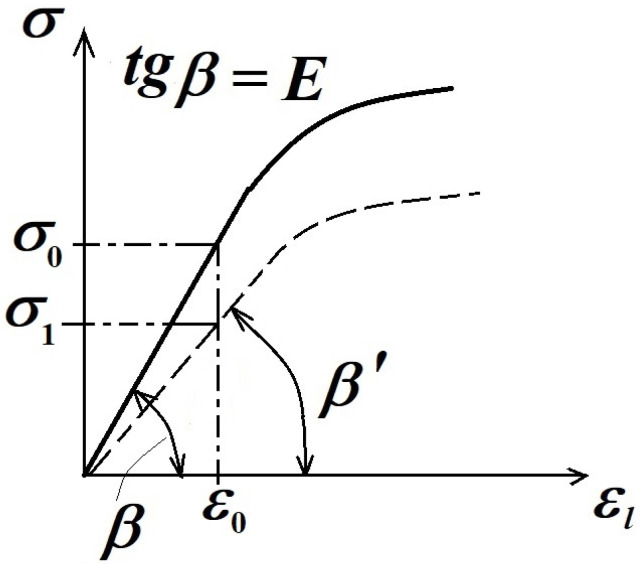
The σ−ε nominal stress–strain curve.

In the hypothesis of the constant cross-sectional area $\left(A=const.\right)$, one can draw up the so-called “nominal σ−ε characteristic curve” (Figure 12); its gradient (slope) will represent the demanded *E* elastic modulus, i.e.,

(8) tg β=E

From Figure 12, one can observe that for a material with lower $E^{\prime }$ elastic modulus, the σ−ε curve is used. For a nominal $\varepsilon_{0}$ strain, instead of $\sigma_{0}$ stress and a lower $\sigma_{1}$ value, i.e., this latter material for a lower applied load (and a corresponding $\sigma_{1}$ stress), it will present the same nominal $\varepsilon_{0}$ strain, which influences, in a negative sense, the load-bearing capacity of this material.

One has to underline that the aforementioned $\varepsilon_{t}$ transversal strain (lateral swelling) during the cyclical mastication loading is responsible for the undesired cyclical loading of the walls (by bending) of the filled tooth, leading to the earlier damage of the filled tooth. This phenomena increase together with the growing up of the ν Poisson’s ratio, which is undesired in dental practice.

Consequently, for an efficient DFMs selection, the most significant mechanical characteristic will be exactly this ν Poisson’s ratio, not the aforementioned and widely (generally) established elastic modulus from the literature.

In the strength of materials, it is a well-known fact that a cyclical loading leads to a weakening of the subjected materials’ mechanical characteristics, more intensively for the anisotropic ones such as the DFMs.

The DFM interacts with the natural tooth material not only during the former’s polymerization (in the case of the composite materials) and its solidification, but during the mastication too. In this sense, one will generate significant internal stresses on the interface of the DFM and the wall of the dental cavity, which can finally produce undesired damage of the filled tooth. The produced shrinkage of the DFM compromises its marginal adaptation at the decay’s wall (the prepared cavity inside a damaged tooth) and, finally, this poor adaptation will lead to micro-leakages, micro-cracks, as well as to secondary caries lesions [42,44]. The existent differences between the elastic moduli as well as the corresponding ν Poisson’s ratios of the DFM and the natural dental tissue represent, in fact, one of the major reasons (cause) for the above-mentioned damages.

As a first attempt of the authors of the present survey, in [44], they describe their preliminary investigations on high-accuracy establishment of the Young’s modulus (elastic modulus) for different DFMs. As an illustration of the described test-methodology [44], here they offer the results of two micro-hybrid composite materials, i.e., Charisma-Heraeus Kulzer, respectively Extra Fil Voco. In order to reach the proposed goal, the authors conceived an original loading device and testing bench. Small manufactured cylindrical samples were subjected to uni-axial compression.

**Figure 13:**
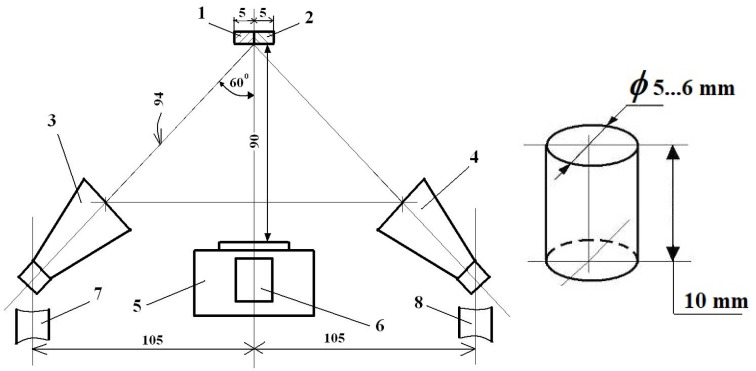
Experimental set-up and dimensions for the specimen tested: 1—reference plate; 2—tested object; 3,4—diodes; 5—Michelson Interferometer; 6—camera; 7,8—the high-stiffened polycarbonate rods.

Figure 13 shows the experimental set-up and the sizes of the small specimen, respectively. The small reference plate 1 (having a width of 5 mm) is appropriately put beside (placed beside) the tested specimen (2). The laser diodes 3 and 4, which assure a good and equal illumination on the observed surface, are fixed onto the high-stiffened polycarbonate rods (7, 8).

The Michelson Interferometer 5 and the four-Mega pixel CCD camera 6 are disposed in a normal direction to the object. The distances are given in mm. A comparison, by the reference plate method, of the image that belongs to the small and unloaded plate to that of the specimen makes possible a good and accurate in-plane strain analysis (see details on Section 2.9, g at ESPIM).

In order to ensure a reliable statistical evaluation, an adequate number of specimens were manufactured (twelve from each sort of *FDM*s).

**Figure 14:**
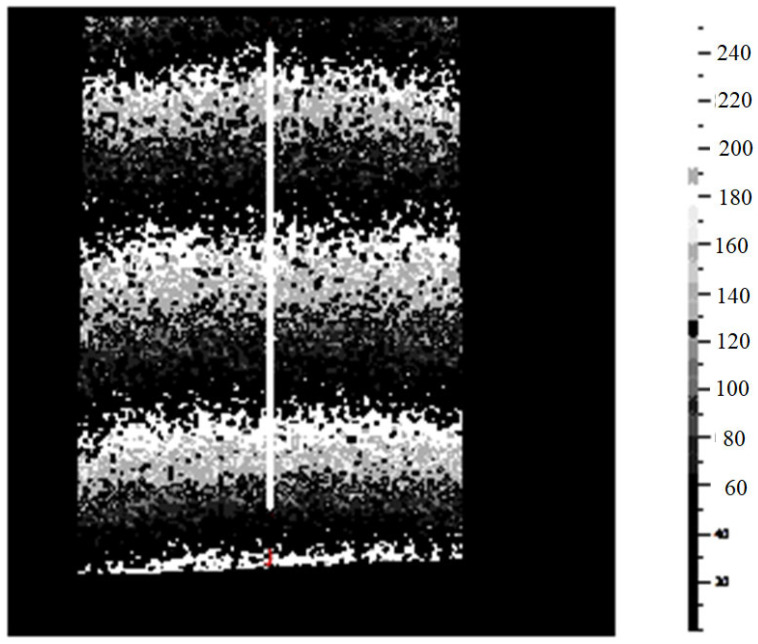
Cylindrical specimen #1, Filtered data.

**Figure 15:**
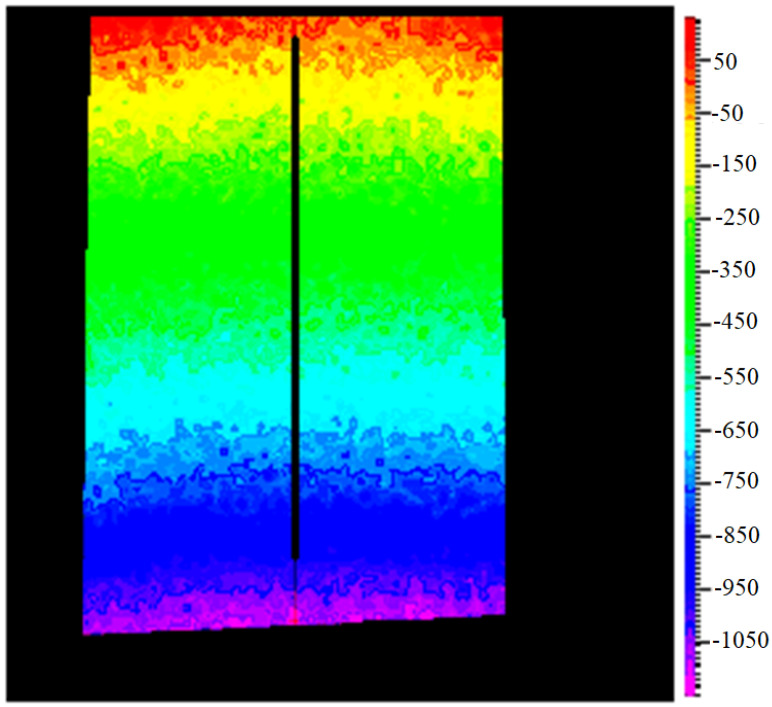
Cylindrical specimen #1, Evaluated data.

Figure 14 and Figure 15 show, for one tested cylinder-shape specimen and for a given applied F load, two of the most important steps in the displacement’s field evaluation: the filtering and the final evaluation along a pre-selected central vertical line (having a known length $l_{0}$), which represents the x-axis of the specimen.

Using the values of the displacements on a selected line (e.g., a central/median line of the specimen, but not only there), one can determine the elongation $\delta_{l}$ and then the corresponding axial (longitudinal) strain $\varepsilon_{l}$ with Equation (5).

By knowing the applied F load and the cross-sectional A area of the specimen, with Equation (7), one can determine the normal (axial) stress σ.

Consequently, one can draw the σ−ε stress–strain curve, from where the sought Young’s modulus can be determined without difficulties, as was shown in Figure 12 and Equation (8).

**Figure 16:**
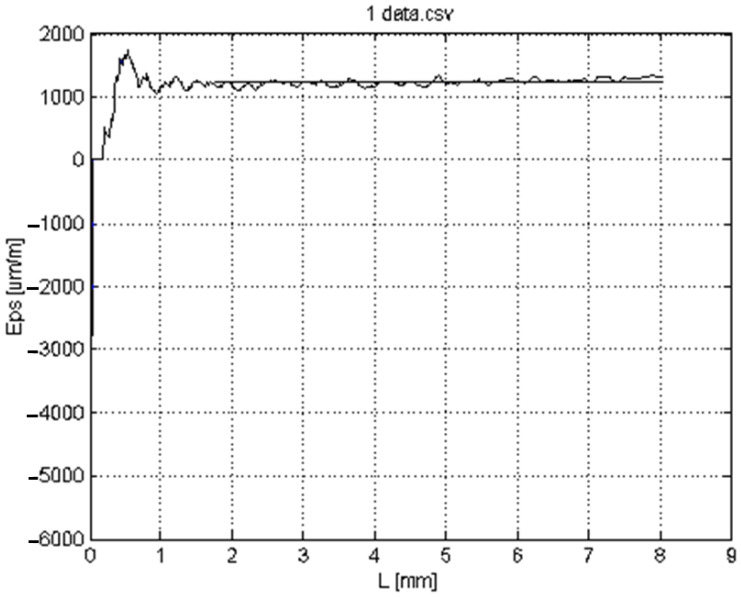
Axial (longitudinal) $\varepsilon_{l}$ strains variation for a given force along the selected longitudinal reference line on the tested specimen.

Figure 16 illustrates the accuracy of the developed strain evaluation procedure. The selected initial length $l_{0}=8\;\mathrm{mm}$ was divided into a very high number of pixels and starting from the approximately the 100th pixel (!), corresponding roughly to 0.4 mm, it became possible to perform an adequate calculation, presented by the zigzag graph.

Taking a mean value (represented by a straight line) of the zigzag graph (starting from the mentioned 100th pixel), finally the mean strain $\varepsilon_{l}=1240.1\;$ μm/m, was obtained, which corresponds to one of the tested specimens’ loading, with a given applied compressive force (load).

Certainly, this procedure, repeated for each load of a given tested specimen, offers both the σ−ε stress–strain curve and the corresponding elastic modulus (Young’s modulus).

For a given type of DFM, i.e., for all identical tested specimens manufactured from a given DFM, by an accurate statistical evaluation of the so-obtained values, we obtained the probable σ−ε stress–strain curve and the corresponding probable $\hat{E}$ elastic modulus (Young’s modulus) too.

**Figure 17:**
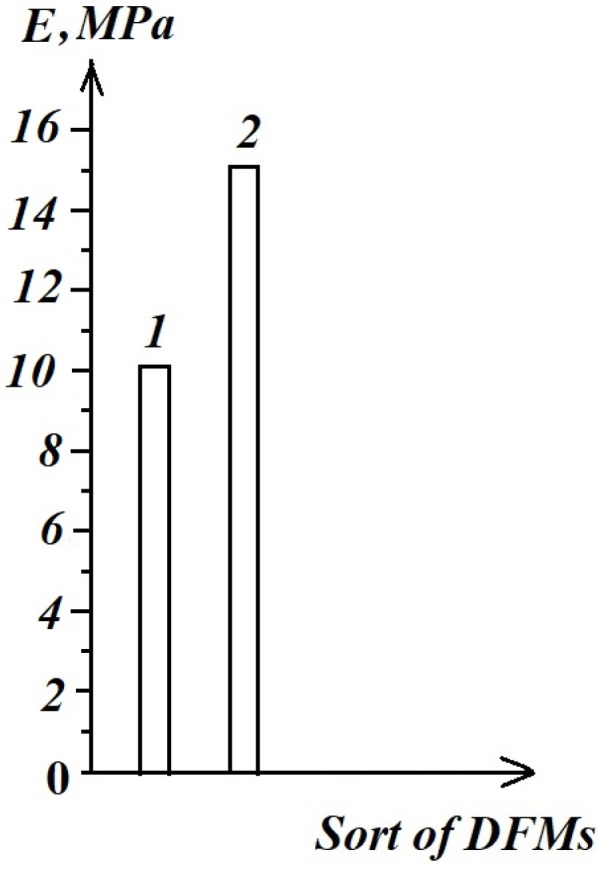
The probable Young’s modulus for the tested DFMs: 1—Charisma-Heraeus Kulzer; 2—Extra Fil Voco.

In Figure 17, a comparative analysis of the probable $\hat{E}$ Young’s modulus for the tested DFMs is shown. The authors obtained the following:

$\hat{E}$ = 10,125 MPa for the Charisma-Heraeus Kulzer;

$\hat{E}$ = 15,228 MPa for the Extra Fil Voco, which is in good correlation with the literature reported results [21], respectively [42].

It is known from the literature that, among the precision indices related to a statistical analysis of the experimental data (therefore of the measurements), the standard deviation has a special significance [45,46,47]. A simple and effective method of statistical analysis of the obtained experimental data is also presented, further applied by the authors. Consider a series of *n* values of the measurements $\left(X_{1},\;X_{2},\ldots \right)$, which present a certain frequency of occurrence $f_{j}$, so that

(9) $$\sum f_{j}=n$$

For this set of measurements, it is desired to determine both the probable average value $\hat{X}$ and the related precision index σ.

Also taking into account the frequency of occurrence $f_{j}$ of the measurements, we will have the following calculation relations:

- Probable average value

(10) $${\hat{X}}^{\prime }=\frac{\sum X_{i}}{n}=\frac{\sum \left[{\bar{X}}^{\prime }+\left(X_{i}-{\bar{X}}^{\prime }\right)\right]}{n}={\bar{X}}^{\prime }+\frac{\sum \left(X_{i}-{\bar{X}}^{\prime }\right)}{n}={\bar{X}}^{\prime }+\frac{\sum f_{j}\cdot \left(X_{i}-{\bar{X}}^{\prime }\right)}{n}={\bar{X}}^{\prime }+\Delta^{\prime }$$

- The square of the standard deviation

(11) $$\sigma^{2}=\frac{\sum x_{i}^{2}}{n}=\frac{\sum {\left(X_{i}-\bar{X}\right)}^{2}}{n}=\frac{\sum {\left(X_{i}-{\bar{X}}^{\prime }\right)}^{2}}{n}-\Delta^{2}$$

Following the calculations, the desired quantities $\left[\Delta^{\prime },\;{\hat{X}}^{\prime },\;{\left(\sigma^{\prime }\right)}^{2},\;\sigma^{\prime }\right]$ will be obtained, which we must verify through a simple procedure, namely, by repeating the previous calculations with the consideration of another value of the predictable average value ${\bar{X}}^{\prime \prime }$. In this case, the calculations will result in the sizes $\left[\Delta^{\prime \prime },\;{\hat{X}}^{\prime \prime },\;{\left(\sigma^{\prime \prime }\right)}^{2},\;\sigma^{\prime \prime }\right]$.

(12) $$\Delta^{\prime }=\frac{a^{\prime }}{n}\;;$$

(13) $${\hat{X}}^{\prime }={\bar{X}}^{\prime }+\Delta^{\prime }\;;$$

(14) $${\left(\sigma^{\prime }\right)}^{2}=\frac{b^{\prime }}{n}-{\left(\Delta^{\prime }\right)}^{2}$$

(15) $$\sigma^{\prime }=\sqrt{\frac{b^{\prime }}{n}-{\left(\Delta^{\prime }\right)}^{2}}\;;$$

In the present case, the measurements that were subjected to statistical analysis referred to the elasticity moduli of the two types of DFMs, i.e., Charisma-Heraeus Kulzer, and Extra Fil Voco.

The strictly metrological conditions for carrying out the measurements should also be mentioned, among which the following can be mentioned:

- A number of 25 samples were made, of which, based on a selection regarding their dimensional deviations, a number of 12 samples were retained;
- Their loadings were carried out with the help of calibrated weights, and the loading order was kept the same;
- The loads, being always located below the limit of proportionality, were repeated three times, each time retaining their rounded average.

In the first case, for Charisma, from the set of twelve tests (samples), one provided values far removed from the rest, which is why it was excluded from the actual statistical analysis. Consequently, here we had n=11.

In the first hypothesis (Table 2), it was accepted that ${\bar{X}}^{\prime }=10,122\;$ [MPa] was the predictable average value when the data below resulted in the following:

$$\Delta^{\prime }=\frac{a^{\prime }}{n}=\frac{31}{11}=2.818182\;;$$

$${\hat{X}}^{\prime }={\bar{X}}^{\prime }+\Delta^{\prime }=10,122+2.818182=10,124.82\;\mathrm{MPa};$$

$${\left(\sigma^{\prime }\right)}^{2}=\frac{b^{\prime }}{n}-{\left(\Delta^{\prime }\right)}^{2}=\frac{191}{11}-{\left(2.8181882\right)}^{2}=9.4214888$$

$$\sigma^{\prime }=\sqrt{\frac{b^{\prime }}{n}-{\left(\Delta^{\prime }\right)}^{2}}=\sqrt{9.424888}=3.069444\;;$$

In the second hypothesis (Table 3), it was accepted that ${\bar{X}}^{\prime \prime }=10,124\;$ [MPa] was the predictable average value when the data below resulted in the following:

(16) $$\Delta^{\prime \prime }=\frac{a^{\prime \prime }}{n}=\frac{9}{11}=0.818182\;;$$

(17) $${\hat{X}}^{\prime \prime }={\bar{X}}^{\prime \prime }+\Delta^{\prime \prime }=10,124+0.818182=10,124.82\;\mathrm{MPa};$$

(18) $${\left(\sigma^{\prime \prime }\right)}^{2}=\frac{b^{\prime \prime }}{n}-{\left(\Delta^{\prime \prime }\right)}^{2}=\frac{111}{11}-{\left(0.818182\right)}^{2}=9.4214888$$

(19) $$\sigma^{\prime \prime }=3.069444\;.$$

Therefore, the statistical analysis was validated and the following rounded values were considered as the final values for DFM Charisma:

(20) $$\hat{X}=10,125\;\mathrm{MPa};\;\hat{\sigma }=3.07\;.$$

Additionally, in the case of Extra Fil Voco, out of the 12 sets of tests (samples) only 11 presented close values, with the last being eliminated from the actual statistical analysis.

Consequently, here too we had n=11.

In the first hypothesis (Table 4), it was accepted that ${\bar{X}}^{\prime }=15,220\;\left[\mathrm{MPa}\right]$ was the predictable average value when the data below resulted in the following:

$\Delta^{\prime }=8.090909\;;$ ${\hat{X}}^{\prime }=15228.09\;\mathrm{MPa};$ ${\left(\sigma^{\prime }\right)}^{2}=29.17355$; $\sigma^{\prime }=3.645847\;.$ In the second hypothesis (Table 5), the above was accepted as the predictable average value, when the data below resulted in the following:

$\Delta^{\prime \prime }=0.090909\;;$ ${\hat{X}}^{\prime \prime }=15228.09\;\mathrm{MPa};$ ${\left(\sigma^{\prime \prime }\right)}^{2}=29.17355$; $\sigma^{\prime \prime }=3.645847\;.$ Therefore, the statistical analysis was validated and the following rounded values were considered as the final values for DFM Charisma:

$$\hat{X}=15,228\;\mathrm{MPa};\;\hat{\sigma }=3.65\;.$$

**Figure 18:**
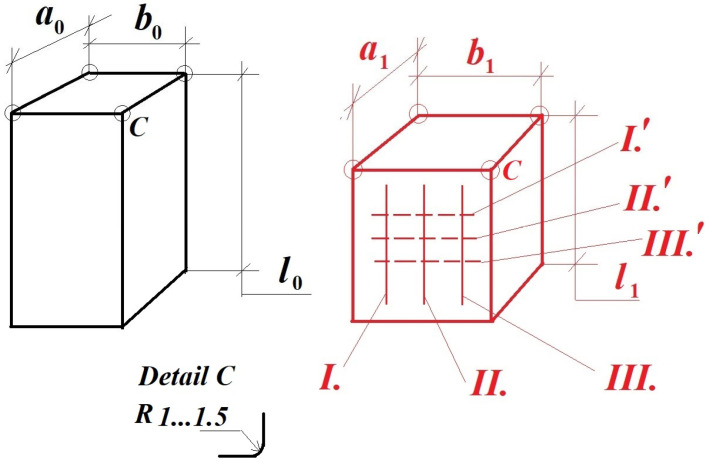
The proposed test specimen for obtaining the Poisson’s ratio: I, II, III—the longitudinal investigations directions; I′, II′, III′—the transversal investigations directions.

Observation: Due to the accuracy of the ESPI method, the previously mentioned protocol, as well as the data acquisition, and the repeatability of the measurements were achieved with good precision. Obviously, in the case of future investigations with the aim of creating a database, where the number of samples subjected to tests will be greater, but also the methodology proposed with the new type of prismatic sample (see Figure 18) will be used, they will be able to obtain values and precision indices even closer to the real case. These data will be able to serve both dentists and those involved in numerical modeling in obtaining optimal results.

Of course, the performed tests can be improved by involving a larger number of tested specimens for a given DFM, as well as by including more kinds of DFMs, in order to obtain an adequate database for dentists.

It is a well-known fact that during the mastication, the filling material is subjected to compression and consequently its lateral shrinkage appears, which is related strongly also to its mechanical characteristics (Poisson’s ratio, Young’s modulus).

Depending on the shrinkage magnitude, the value of the horizontal load will produce a cyclical bending of the lateral wall of the tooth, which will be different in different cases. Taking into consideration that the influence of the ν Poisson’s ratio is most significant in this phenomenon, consequently, a lower Poisson’s ratio produces a lower cyclic loading and the corresponding filling material will be more adequate for the dental practice.

Using these differences, it is possible to evaluate and decide what filling material should be preferred or used.

The above-presented experimental investigations represent in fact the first step of the authors in finding the most efficient way to select the optimal DFM.

Based on the performed tests’ results, the involved dental specialists from our region intend to include in their further practice the proposed approach, and will contribute to creating an adequate database with the most-applied DFMs’ mechanical characteristics, mainly their Young’s modulus and Poisson’s ratio. Even at this moment, it is quite difficult to create such a database, but they hope that with a regional co-operation, their effort will become successful; regarding the DFMs’ manufacturer companies, there are only a few options for helping this effort.

## 5. Conclusions

Based on the obtained results and practice, the authors of this survey, in the forthcoming period, propose similarly to obtain the desired ν Poisson’s ratio too.

In this sense, they propose a new type of specimen, a rectangular-prismatic one (Figure 18) with rounded corners, as is indicated in Figure 18, detail *C*.

The rounded corners are necessary in order to avoid the inadequate material density in these narrow zones. This kind of specimen assures a better analysis of the longitudinal shortening and well as the transversal swelling compared to the cylinder-shaped ones. One has to take into consideration that all specimens are manufactured from anisotropic materials, having in each point of them different mechanical characteristics.

One can observe from Figure 18, which became possible here, that for every tested specimen, taking for analysis a minimum of three longitudinal and transversal directions (selected lines) in order to perform a better statistical evaluation of the $\varepsilon_{l},\;\varepsilon_{t}$ strains, the Young’s modulus of the corresponding ν Poisson’s ratios need to be calculated. In this case, the involved numbers of the statistically required specimen, as well as their testing time, will be strongly diminished, as well as the involved time and tested specimens too.

Finally, by combining all of these results, i.e., *I*-*I*′; *II*-*II*′; *III*-*III*′; *I*-*II*′; *I*-*III*′; *II*-*I*′; *II*-*III*′; *III*-*I*′; *III*-*II*′, one can obtain the most probable values for the above-mentioned parameters, i.e., $\hat{E},\;\hat{\nu }$.

The following significant conclusions can be drawn:

The experimental determination of the mechanical characteristics, mainly the E elastic modulus and the ν Poisson’s ratio, represents a very important step in the optimal selection of the proper (most suitable) DFM;

In this sense, the authors do not recommend the above-presented three-points as well as the four-points bending test results. Instead, they suggest the illustrated ESPI/shearography method, which surely offers more closer and credible (realistic) results for these parameters;

The involved experimental methods have to be, as much as possible, full-field and non-contact ones; the best method at this moment, based on the authors’ best knowledge, is ESPI/Shearography;

The higher the ν Poisson’s ratio of the involved DFM is, the greater the responsibility of diminishing the filled tooth’s lifetime;

Generating an adequate database (considering the nowadays most involved DFMs) with the ν Poisson’s ratios and the E elastic moduli, not only for dental practice but also for the further numerical simulations by the FE Method, will represent a very useful starting point;

The authors of the present survey intend in the forthcoming period to start a searching examination of the nowadays widely used DFMs.

The authors also express their hope that this survey will be useful both for dentists as well as for the FE’s users.

The *FE* users will obtain closer results in their numerical simulation if they consider, instead of the existent *FE* databases, the test results of such ESPI/Shearography tests for a given DFM. In this sense, one has to mention that in these *FE* databases, the mechanical characteristics are introduced as some global values of the involved $\left(E,\;\nu \right)$, established usually by means of three-point and four-point bending tests, which are not recommended from the strength of materials point of view.

The global values rarely represent the given DFM’s characteristics, and, unfortunately, the companies are not very interested in publishing these real values, corresponding to the achieved packet of DFMs.

## Figures and Tables

**Table 1:** Synthesis of the analyzed investigation methods.

Investigation Method	Advantages	Limits	Observations/Remarks
Electrical strain gauge (ESGM) [1,2,3,4,5,6,7,8]	High-accuracy method: its accuracy is inversely proportional to the active length of the strain gauge	Offers information only related to a given point and given grid orientation; it is a material-selective method, i.e., both the strain gauges and glues are strongly dependent on the tested object’s material; not very suitable for small specimen testing, mainly such manufactured from materials with a high gradient of mechanical characteristics	Does not offer global information
Thin Photo-elastic Layer’s Technique (PSM) [1,3,4,5,6,7,8,13]	Full-field method: offers the stress-state of the covered surface with the photo-elastic layer	Being a direct contact method, the correctness measures of the obtained stresses are strongly influenced by the applied layer’s thickness due to the stiffness changing; its accuracy depends on the applied layer’s thickness (the accuracy is inversely proportional to the layer’s thickness); its accuracy is much lower than the other approaches from this synthesis	Presents a relatively low accuracy due to the fact that a minimum 4…5 fringes are necessary to obtain an adequate stress-state evaluation; this number of fringes directly depends on the applied load magnitude as well as the adequate thickness of the photo-elastic layer, which in the case of these small specimens is very difficult to obtain
Moiré-fringe method (MFM) [1,3,4,5,6,7,8,14,15,16,17]	Full-field method: offers the displacement, and the strain field with an adequate accuracy, mainly for the relatively large tested surfaces; it is a universal testing method; the same equipment can be applied for large sorts of materials;	The applied grid’s pitch determines the accuracy of the method; in the case of the small specimens, it requires very high density grids; its accuracy is much lower than the holographic interferometry, ESPI, respectively, and the VIC/DIC methods	
Holographic Interferometry (HIM). [3,4,5,6,7,9]	It is a very high-accuracy investigation method; it is a non-contact and full-field method; it is a universal method (the same equipment can be applied for large sorts of materials, even isotropic, orthotropic, or anisotropic ones);	It requires a strong vibration insulation; the common approach supposes a maximal displacement lower than λ/2, where λ represents the lighting source wavelength; the fringe evaluation is a relatively difficult and time-consuming activity; it requires highly-qualified persons as well as expensive consumables; nowadays, it is recommended mainly only for basic research activities, where the invested large amount of money can be justified	The maximal λ/2 displacements requirement in this case it is not a real disadvantage, taking into consideration the applied load and the corresponding displacements
Brittle Coating (BMC) [1,4,5,6,7,8]	It is a full-field method; even though it is a direct contact method, the applied very thin special varnish (lacquer) does not influence the object’s stiffness; it offers the stress field of the covered surface; it does not involve highly-qualified persons	It is a relatively low accuracy method; usually, it is applied as a preliminary investigation method in order to put in evidence the main stresses loci and their directions; it is recommended for the objects having relatively large surfaces	
Video Image Correlation (VIC) or Digital Image Correlation (DIC) method [5,11,12]	It is a powerful, noncontact and accuracy full-field method; it can be applied also in working/industrial conditions because the vibration’s effects are eliminated by software; the same equipment can be applied for a large variety of materials and for large displacement intervals, starting from microns up to several centimeters	It has a little bit lower accuracy than the holographic interferometry and the ESPI; the equipment is relatively expensive	Its 3D Micro-DIC version offers a much higher accuracy
Electronic Speckle Pattern Interferometry Method-ESPI (ESPIM) [3,5,9,10]	It is a very powerful, noncontact and high-accuracy full-field method; it can be applied also in working/industrial conditions because the vibration’s effects are eliminated by software; the same equipment can be applied for a large variety of materials;	It has a little bit lower accuracy than the holographic interferometry; the equipment is relatively expensive. It supposes a relatively small displacement field, with the maximal λ/2 displacements requirement, where λ represents the lighting source wavelength.	It is fully recommended by the authors for the mentioned investigations. Its initial limitation in displacement is superable/overcome by the “cascading” technique [5,7,23] Due to the small investigated surfaces as well as the applied small loadings, this disadvantage does not represent a limitation of its involvement.

**Table 2:** The predictable average value in the first hypothesis (Charisma).

$X_{i}\;$ **[MPa]**	$f_{j}$	${\bar{X}}^{\prime \prime }=15,228\;$ **[MPa]**		
		$X_{i}-{\bar{X}}^{\prime }$	$f_{j}\cdot (X_{i}-{\bar{X}}^{\prime })$	$f_{j}\cdot {\left(X_{i}-{\bar{X}}^{\prime }\right)}^{2}$
15,217	1	−11	−11	121
15,222	2	−6	−12	72
15,228	3	0	0	0
15,232	4	4	16	64
15,236	1	8	8	64
∑:	n=11		$a^{\prime \prime }=1$	$b^{\prime \prime }=321$

**Table 3:** The predictable average value in the second hypothesis (Charisma).

$X_{i}\;$ **[MPa]**	$f_{j}$	${\bar{X}}^{\prime \prime }=15,228\;$ **[MPa]**		
		$X_{i}-{\bar{X}}^{\prime }$	$f_{j}\cdot (X_{i}-{\bar{X}}^{\prime })$	$f_{j}\cdot {\left(X_{i}-{\bar{X}}^{\prime }\right)}^{2}$
15,217	1	−11	−11	121
15,222	2	−6	−12	72
15,228	3	0	0	0
15,232	4	4	16	64
15,236	1	8	8	64
∑:	n=11		$a^{\prime \prime }=1$	$b^{\prime \prime }=321$

**Table 4:** The predictable average value in the first hypothesis (Extra Fil Voco).

$X_{i}\;$ **[MPa]**	$f_{j}$	${\bar{X}}^{\prime \prime }=15,228\;\left[\mathrm{MPa}\right]$		
		$X_{i}-{\bar{X}}^{\prime }$	$f_{j}\cdot (X_{i}-{\bar{X}}^{\prime })$	$f_{j}\cdot {\left(X_{i}-{\bar{X}}^{\prime }\right)}^{2}$
15,217	1	−11	−11	121
15,222	2	−6	−12	72
15,228	3	0	0	0
15,232	4	4	16	64
15,236	1	8	8	64
∑:	n=11		$a^{\prime \prime }=1$	$b^{\prime \prime }=321$

**Table 5:** The predictable average value in the second hypothesis (Extra Fil Voco).

$X_{i}\;$ **[MPa]**	$f_{j}$	${\bar{X}}^{\prime \prime }=15,228\;\left[\mathrm{MPa}\right]$		
		$X_{i}-{\bar{X}}^{\prime }$	$f_{j}\cdot (X_{i}-{\bar{X}}^{\prime })$	$f_{j}\cdot {\left(X_{i}-{\bar{X}}^{\prime }\right)}^{2}$
15,217	1	−11	−11	121
15,222	2	−6	−12	72
15,228	3	0	0	0
15,232	4	4	16	64
15,236	1	8	8	64
∑:	n=11		$a^{\prime \prime }=1$	$b^{\prime \prime }=321$

## Data Availability

Not applicable.
